# Improving hand hygiene of young children with a digital intervention: a cluster-randomised controlled field trial

**DOI:** 10.1038/s41598-024-56233-9

**Published:** 2024-03-14

**Authors:** Joanna Graichen, Carlo Stingl, Anni Pakarinen, Riitta Rosio, Kirsi Terho, Sebastian A. Günther, Sanna Salanterä, Thorsten Staake

**Affiliations:** 1 Department of Information Systems and Applied Computer Sciences, University of Bamberg, Bamberg, Germany; 2 Department of Nursing Science, University of Turku, Turku, Finland; 3 Turku University Hospital, Turku, Finland; 4 Department of Management, Technology and Economics, ETH Zurich, Zurich, Switzerland

**Keywords:** Disease prevention, Paediatric research

## Abstract

Contagious diseases that affect young children place a great burden on them and their families. Proper hand hygiene is an important measure to reduce the disease burden, however, its implementation in day care centres is challenging. This paper introduces a digital intervention to support independent and good handwashing among young children. The intervention leverages animated instructions triggered by water and soap use, together with a symbolic reward shown to children on a screen during and immediately after handwashing. We tested the intervention in a pre-registered, cluster-randomised controlled field trial in 4 day care centres in Finland and Germany with 162 children over 42 days. The intervention increased soaping time, used as a proxy for handwashing quality, by 5.30 s (+ 62%, p < 0.001). The effect occurs immediately at the onset of the intervention and is maintained throughout the intervention phase.

## Introduction

Even though child mortality is low in industrialised countries^1^, most children are affected by several diseases every year: 93% of children between 3 and 6 years suffer from at least one respiratory infection per year, and 59% have at least one gastrointestinal infection^2^. For children that attend day care centres the number of disease episodes is especially high^3^. The controversial argument that those day care diseases have some positive effects (e.g. by conferring some immunity to school-acquired infections^4,5^) is refuted considering that infections in early childhood often require treatment with antibiotics^6^ and are more likely to be life-threatening than those later in childhood (the severity of infections in children is lowest at school-age^7^). Due to the large number of disease episodes, children suffer not only physically; social development and education are also jeopardised by absenteeism^8^. In addition, families experience hardship from the multitude of disease episodes in children. The family’s daily routines are disrupted to focus on the sick child and the childs care, and family members, especially siblings, might also become infected. Parents face professional disadvantages, and some parents stay away from work altogether, driven by the actual problem of not finding a caregiver when their child is sick^9^. Overall, the costs caused by childhood illnesses are high, not only for affected children and their families but also for society as a whole^10^.

Relatively simple measures exist that can considerably reduce the spread of diseases in day care centres. A particularly effective one is proper hand hygiene: handwashing is a proven measure to decrease the transmission of infectious diseases^11,12^ and can thus reduce sick days of children^13^. Moreover, good hand hygiene practices can already be performed by young children^14^, and teaching children how to wash their hands is beneficial besides the immediate health impact: health-related habits are often formed in early childhood and maintained into adulthood^15,16^. Teaching children good hand hygiene is therefore a promising way not only to improve handwashing hygiene in day care centres^17^, but also to increase low hand hygiene compliance rates among adults^18^ in the future.

Given the importance of hand hygiene for public health, it is not surprising that low handwashing compliance led to various measures that attempt to improve the situation. A measure that became ubiquitous at the latest during the SARS-CoV-2 pandemic is the placement of stickers near washbasins reminding people that handwashing is essential and illustrating how to wash hands properly. However, even among adults, such information campaigns have been shown to have little impact^19^. Another popular measure is personal hygiene training, which offers direct and person-specific feedback. Personal hygiene training has an immediate impact, but is expensive, difficult to scale, and the impact wears off quickly^20^. Recently, camera-based handwashing monitoring systems have been introduced^21^, but their adoption is limited to operation theatres in hospitals due to their high technical complexity and cost.

Studies in different fields have shown that feedback is a powerful tool to alter behaviour (e.g., in the environmental^22^ and health^23,24^ domain). With the advent of digital technologies, more and more (real-time) feedback interventions are emerging^25^. Real-time feedback that is given while an action is being performed and at the point of action, so that a direct response of the individual is possible, has proven to be effective to influence human behaviour and enable habit change^26^. However, most studies on feedback refer to adults and therefore only allow limited conclusions to be drawn for children. E.g., a review of digital feedback interventions featuring 50 interventions includes only two for children^25^. To extend research on feedback interventions for children, we develop a digital intervention for children, based on existing knowledge on feedback interventions and theories about children's learning, such as social cognitive theory^27^. The developed digital system aims at improving the handwashing of 3- to 6-year-old children in day care centres. The overall aim of our study is to explore how handwashing behaviour of young children can be enhanced through a practical and scalable digital intervention.

## Materials and methods

We conducted an eight-and-a-half-week field study in day care centres in Finland and Germany. The study is implemented as a cluster-randomised controlled trial, i.e., day care centres rather than independent individuals are randomly allocated to the control and the treatment group. All subgroups (hosting children between 3 and 6 years old) of a single day care centre belong to one experimental group. This is to avoid information spill over that one has to expect when otherwise children, parents or educators of two neighbouring groups ask questions, discuss, and exchange information in everyday day care centre life. The experiment and used methods are described in the following.

### Participants and randomisation

**Figure 1:**
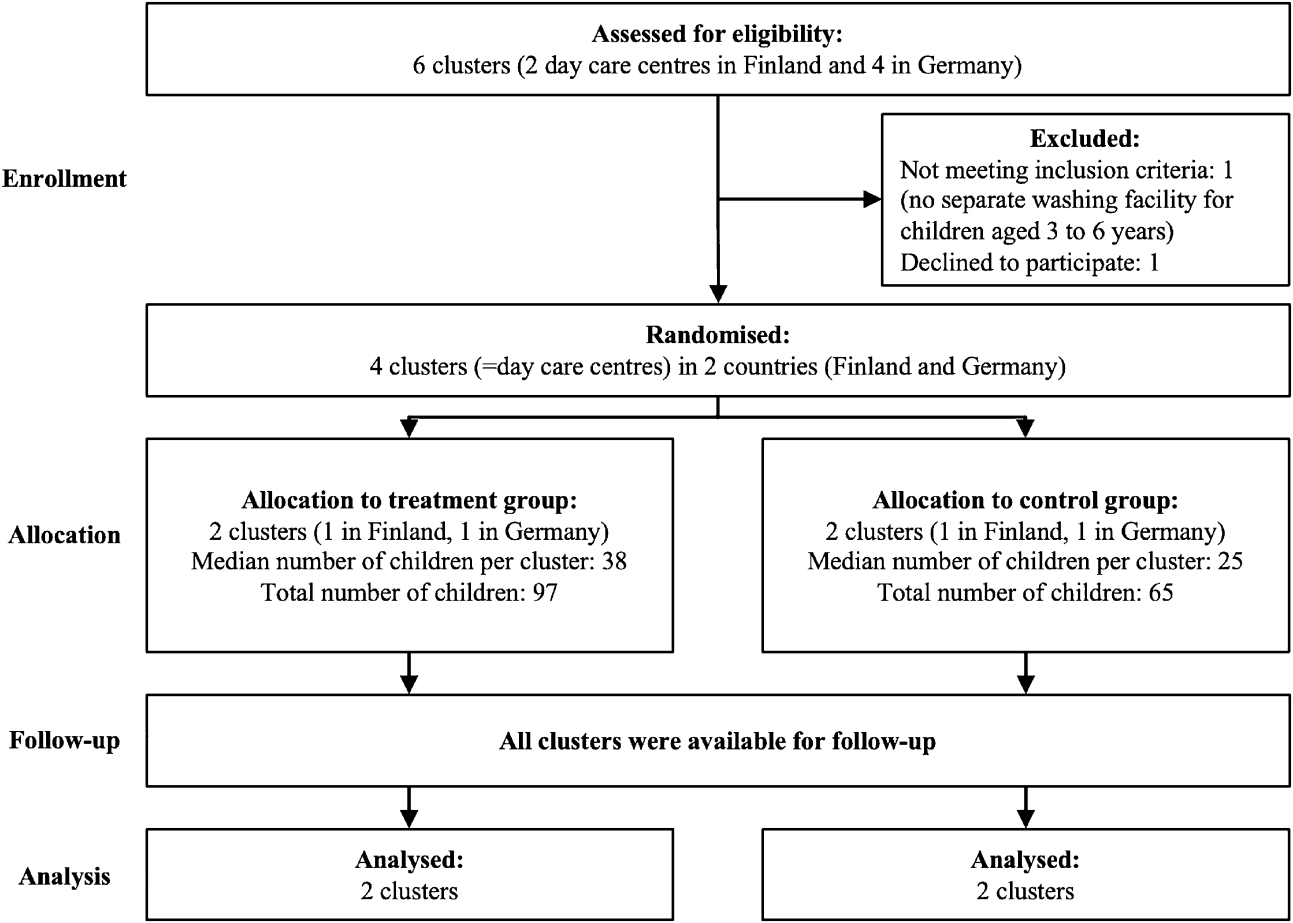
CONSORT flowchart—the flow of clusters through the study from enrolment of day care centres to analysis of data.

Participating day care centres were recruited in Finland and Germany. To be eligible for the study, day care centres had to host children ages three to six in separate groups from children of other ages, and there had to be a dedicated washroom for children from 3 to 6 years old. There were no other eligibility criteria for the day care centres or the individual participants. Two day care centres in Finland and two in Germany were recruited (see Fig. 1). The 2 day care centres in Finland lie in the same region in the southwest, 10 min drive apart, in cities with 35,000 and 7000 inhabitants, respectively. The 2 day care centres in Germany lie in the same city with 80,000 inhabitants in Bavaria. When selecting day care centres, we ensured that none of them was in neighbourhoods with extreme (high/low) income. One day care centre in each country was randomly assigned by a computer program to the treatment condition, while the other was assigned to the control condition.

Day care centre teachers and parents were informed that non-personal handwashing data (anonymous and not attributable to individual children) would be collected in the day care centre as part of a scientific study to improve handwashing. The information also included an indication that specific measures would be taken to improve handwashing in the day care centre. No further details about the treatment were provided, as treatment allocation occurred after informing and recruiting day care centre staff, children, and their parents. Parents, children, and day care centre staff did not know whether they were part of the treatment group or not for the duration of the study.

The information package regarding the study was sent out to the parents via e-mail by the day care centre management and the parents’ council in Germany and via an administration and communication platform used in day care centres and schools in Finland. Handwashing data were collected from all children in the day care centre group because the washbasins are used by all children equally.

### Materials

**Figure 2:**
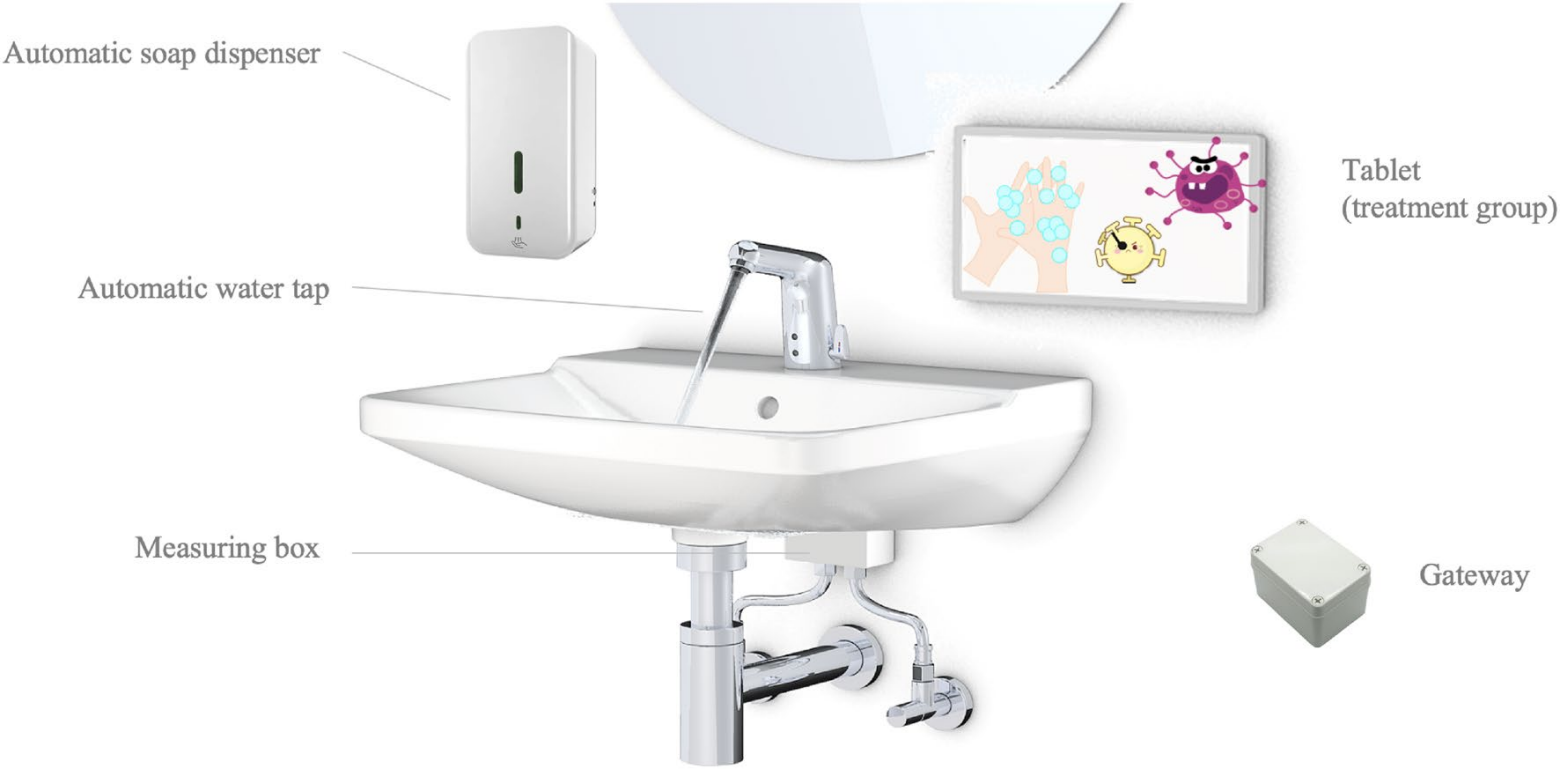
Schematic installation of the digital intervention at one washbasin, including an automatic.

We equipped all handwashing facilities of the bathrooms of the participating day care centres with study hardware (automatic water taps and automatic soap sensors with a digital measuring unit, and gateways), for a schematic installation see Fig. 2. The system connects several sensors with tablets above washbasins in day care facilities that can display real-time interventions to children. Digital sensors, non-visible to the users, measure the flow rate, temperature, time, and duration of each water extraction and the timestamp for each soap extraction. Sensor data is sent via Bluetooth to a gateway that we installed in each bathroom near the ceiling and out of reach for children. The gateway collects the data and relays it to the study’s server infrastructure. Additionally, we equipped the treatment group day care centres at the start of the intervention phase with displays (low-cost tablet PCs with an Android operating system) next to each washbasin that can also communicate with the gateway. The smart soap dispensers and gateways were developed and built by the researchers themselves. A supporting company supplied and installed the smart water taps (see the Acknowledgements section). Prior to that, the hardware had been tested over several weeks in a test setup at a university and several hospitals. Further information on the study hardware is published in Stingl et al.^28^.

### Treatment

**Figure 3:**
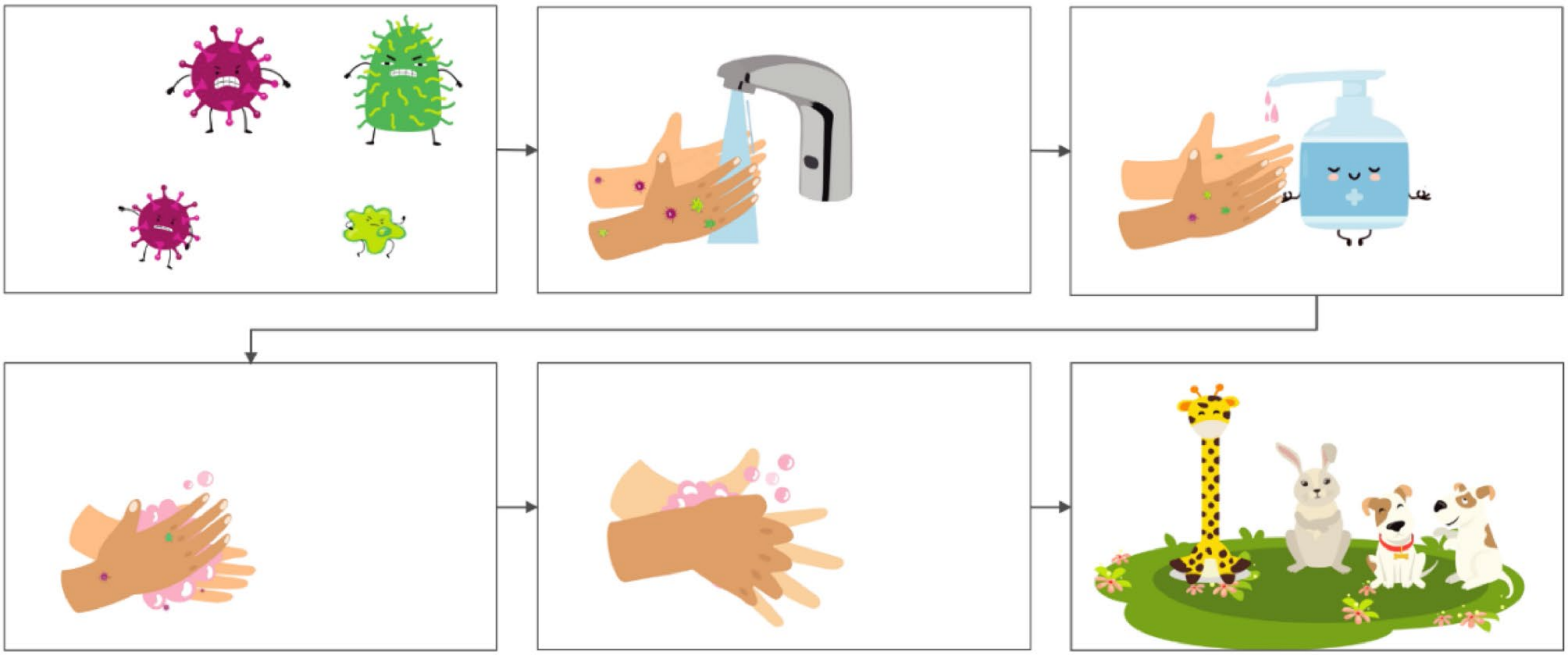
Selected screens of the intervention animation shown on a display above the washbasins in day care centre. Screens include viruses as the screensaver (top row, left), wetting hands (top row, middle), taking soap (top row, right), different soaping movements (bottom row, left and middle) and animals as performance contingent reward (bottom right).

During the intervention phase, while children were washing their hands, they were exposed to a digital teaching element on handwashing on the display next to the washbasin: animated handwashing instruction on the tablet (see Fig. 3 for selected screens). When no one was using the washbasin, the tablet displayed a screen with animated viruses in idle mode. As soon as a child started washing her hands by either extracting water or soap, the handwashing instructions appeared. On subsequent screens, all relevant handwashing steps^29^ were shown: first hands are wetted with water from the tap, then soap is extracted, five different soaping movements are performed, soap is rinsed with water, and hands are dried. Teaching children soaping techniques rather than solely focusing on the duration has been proven successful in prior research^30^. The total length of the animation is 30 s, including 18 s of soaping movements (excluding the idle and the reward screen). If a child took water and soap and waited until the whole animation has been played, it saw merrily dancing animals on the display as a performance-contingent symbolic reward. We deliberately chose animals as the reward because young children place a high moral standing on animals^31^ and their reactions to animals are driven by joy, curiosity, and (for some animals that we excluded from our animation) fear^32^. The intervention was pre-tested before the start of the study with seven children between 3 and 6 years.

### Experimental procedures

**Figure 4:**
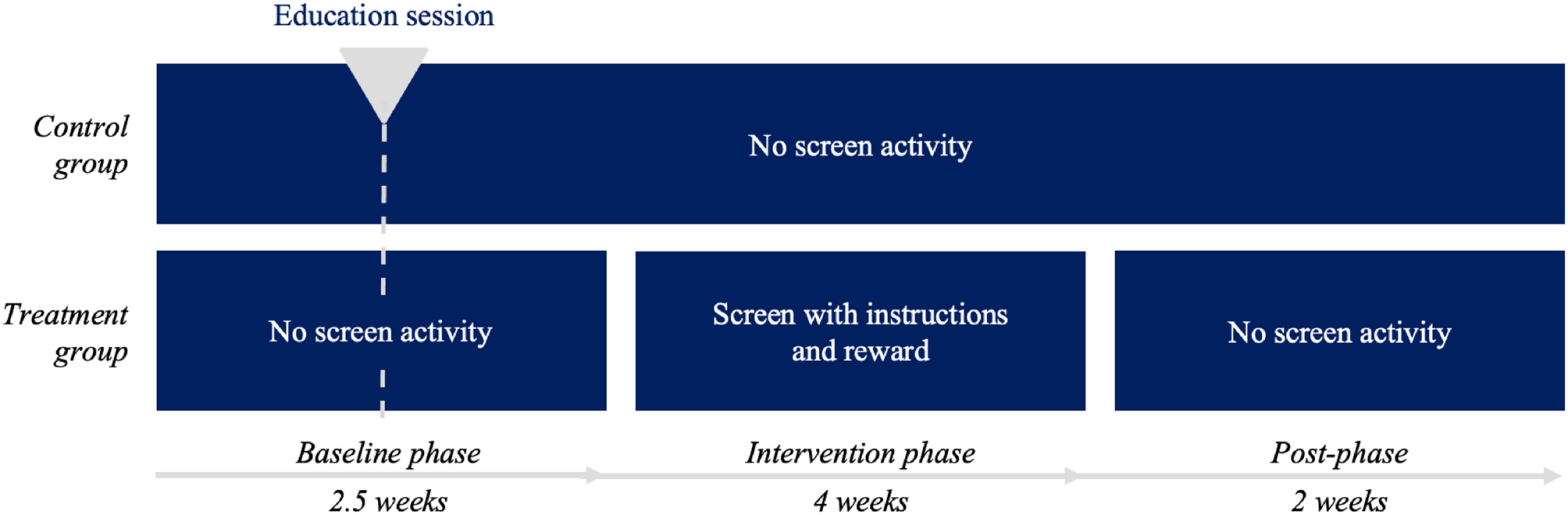
Overview of the experimental setup of the randomised controlled trial with two groups (control and treatment) and three phases (baseline, intervention, and post-phase).

Before the study start, we equipped all 16 washbasins in six bathrooms of the four participating day care centres with the study hardware (automatic water taps, automatic soap dispensers and gateways; no tablets yet). While some day care centres were already equipped with automatic water taps and/or soap dispensers before our study, others did not have automatic equipment. Installing the study hardware well before the study started, ensured that children of all day care centres were able to use the automatic water taps and soap dispensers independently and intuitively. The tablets were installed above the washbasins in the treatment day care centres at the start of the intervention phase. Figure 4 gives an overview of the experimental setup of the randomised controlled trial. The overall time frame for data collection was limited by the day care centres’ closing times due to public holidays and vacations. The three experimental phases were divided among this time frame (with a focus on the intervention phase). With the start of the two-and-a-half-week baseline phase, the hardware devices began measuring handwashing data in the participating day care centres. In the middle of the baseline phase, day care centre teachers showed an educational video (based on the handwashing recommendations from the Centers for Disease Control and Prevention^33^) on proper handwashing to all children both in the control and the intervention day care centres. The children watched the video several times in small groups, which lasted about one and a half minutes. This aimed to ensure that children across groups had the same knowledge of how proper handwashing is performed.

With the start of the 4-week intervention phase, treatment group day care centres were equipped with tablets. Neither children, parents, nor day care centre staff of the control group was informed about the existence of the screens in the treatment day care centres. The 2-week post-intervention phase started right after the tablets were uninstalled in the treatment day care centres.

### Outcomes

The study’s primary outcome is the intervention’s effectiveness, measured by the impact on the soaping time, which in turn is used as a proxy for overall handwashing quality. Guidelines for handwashing from health authorities require soaping hands for about 20 s^33,34^, since increasing soaping time reduces bacteria count^35^. Thus, soaping time is a prerequisite and indicator of a good handwashing quality. Soaping time in our study is defined as the duration between the extraction of soap from the dispenser and starting the water at the tap again to rinse off the soap. Handwashing processes in our study are defined as soap extractions that are followed by water extractions after soaping. Beyond affecting the handwashing quality, it is conceivable that also the handwashing frequency changed. Thus, handwashing frequency is a secondary outcome measure. Furthermore, we collected additional control variables and measurement data for data sanity checks, including headcount of children per day and day care centre. This allowed us to account for fluctuations (due to sickness, individual holidays, etc.) in the evaluation of the handwashing frequency (number of water extractions per child and day).

### Sample size calculation

The sample size needed to detect a statistically significant difference between the treatment and the control group that we calculated before the experiment with a power test is n = 123 children. Calculation details for the power test are described in the subsequent section.

Following Cohen’s power analysis for multiple regression, the sample size n for individually randomised samples results in 31, when using a power of 0.80, a significance criterion of 0.05, and a large effect size (Cohen’s f^2^ of 0.35) as a prerequisite^36^. However, randomising by cluster leads to some power loss compared to randomisation on an individual level^37^. The power loss can be quantified by the ratio of the number of subjects required in the cluster trial versus the number of subjects needed using individual randomisation, the so-called design effect^38^. With a calculated design effect of D = 3.958 (calculation see below), this ultimately results in a sample size for the cluster-randomised sample of 123 children or (with an assumed group size of 30 children per day care centre) 4 day care centres.

Estimating the design effect requires the estimation of the intracluster correlation coefficient (ICC). From a study investigating ICCs for educational institutions to study academic outcomes, we chose the most appropriate ICC reported for day care centres, which is 0.102^39^. With an estimated day care centre group size of m = 30 children, we calculated the design effect D to be

1 $$D=1+\left(m-1\right)*ICC=1+\left(30-1\right)*0.102=3.958$$

### Statistical methods

We hypothesised that the handwashing quality, as expressed by soaping time and measured by our system, would significantly increase in the treatment group. To test this hypothesis, we pre-processed the data that was collected by our system. Implausible data points were removed, e.g., water extractions recorded outside of day care hours due to cleaning. In a second step, we examined the imbalances of the clusters and calculated the ICC, resulting in 0.06. To ultimately evaluate the effects of our intervention, we used a linear fixed-effect regression model for panel data to estimate the relationship between the dependent variable soaping time, the treatment condition, the intervention phase, and the post-phase. The dataset allows us to analyse soaping time as the dependent variable. The analysis was carried out at the day care centre level rather than at a water tap level. However, water tap-level data supports the significant results that we found. Since all children in the day care centres use the washbasins that are modified with our study hardware, we include all cluster members in our data measurement and analysis and thus ensure unbiased estimates of the intervention effect. We considered a p value of 0.05 as significant in all our analyses. The statistical analyses were performed with R version 4.0.2 (2020-06-22).

To formally estimate the results, we model the following relationship using ordinary least squares:

2 $${y}_{it}={a}_{i}+{IN}_{it}^{intervention}\times \left({\beta }_{1}+{\beta }_{2}{T}_{i}\right)+{IN}_{it}^{post}\times \left({\beta }_{3}+{\beta }_{4}{T}_{i}\right)+{\varepsilon }_{it}$$

where the dependent variable ${y}_{it}$ represents the soaping time in day care centre *i* on timestamp *t*. We include an individual fixed effects coefficient ${\alpha }_{i}$ for each day care centre to control for fixed differences in the washing places across the different day care centres. The variable ${IN}_{it}^{intervention}$ is 0 during the baseline and the post-phase, and 1 in the intervention phase. By contrast, the variable ${IN}_{it}^{post}$ is 0 during the baseline and the intervention phase, and 1 in the post-phase. ${T}_{i}$ is a treatment group indicator that takes the value of 1 if a day care centre belongs to the treatment group and is else 0. The standard errors are clustered on the day care centre level. The error term ${\varepsilon }_{it}$ captures all effects that are not considered in our model.

To analyse changes in the handwashing frequency, we again use Eq. (1). Here, the normalised number of handwashes per day is the dependent variable (i.e., normalised by the specific number of cared children in the day care centre on a given day).

### Ethical approval

The ethics committees of the University of Turku and the University of Bamberg approved the study. Furthermore, we obtained a research permit from the authorities of the City of Kaarina, Finland (registration number NRO75/2020) and registered the study as a clinical trial in the ClinicalTrials.gov database (ID NCT04773288; initial release February 2021). All research was performed in accordance with relevant guidelines and regulations. Our digital system collected data for all children present in the day care centre; data collection was anonymous and independent of individual children. However, we asked for written informed consent to interview children, their parents, and the day care centre teachers during the experiment.

## Results

We collected 13,466 handwashing observations (soap extractions with subsequent water extractions) between April and July 2021. In each day care centre, we collected data on at least 42 weekdays. Detailed results are further described below.

### Characteristics of study participants and groups

162 children participated in our experiment. We contacted six eligible day care centres in the first quarter of 2021. Suitable day care centers were administered to participate in the experiment and randomised. No dropouts occurred—all day care centres participated until the end of the study and are thus included in the outcome analysis. On average, our system recorded handwashing activities of 127 children in 4 day care centres over 42 days.

Table 1 provides measured baseline characteristics for the full sample and each cluster, stratified by country. The table reports cluster means and standard deviations for key handwashing characteristics during the baseline phase. The mean soaping time per water tap in the treatment group and the control group do not statistically differ during the baseline phase when there is no tablet present (p = 0.15). The mean soaping time in the baseline phase across the clusters was 8.45 s. Over the course of the experiment (all three phases), the equipment recorded 42,187 water extractions which can be translated into 13,466 handwashing processes.

**Table 1:** Descriptive statistics measurement data, including number of children, and water extraction data for the full sample, per stratum, and per experimental group (control and treatment).

	Full sample	Stratum 1: Finland		Stratum 2: Germany	
		Control group	Treatment group	Control group	Treatment group
*Mean number of children in day care centre per day*^a^	36.60 (9.12)	31.20 (3.98)	41.70 (4.93)	21.56 (4.03)	41.07 (7.09)
*Mean baseline water extractions per day*	248.37 (115.12)	344.84 (55.26)	200.31 (77.71)	108.85 (28.39)	339.46 (61.33)
*Mean baseline water extractions per day and child*	7.71 (2.34)	9.97 (1.01)	5.26 (0.85)	6.20 (1.68)	9.35 (1.34)
*Mean baseline soaping time*	8.45 (7.79)	6.77 (6.84)	7.93 (7.29)	9.05 (8.15)	9.22 (8.12)
*N water extractions*^a^	42,187	12,933	7732	5366	16,156
*N handwashing processes*^a^	13,466	2927	2451	1815	6273

Descriptive statistics for the full sample and the different clusters, based on measurement data from the day care centres. Standard deviations are reported in parentheses.

^a^Reported figures include baseline, intervention, and post-phase.

### Effect of the intervention on handwashing quality and frequency

**Figure 5:**
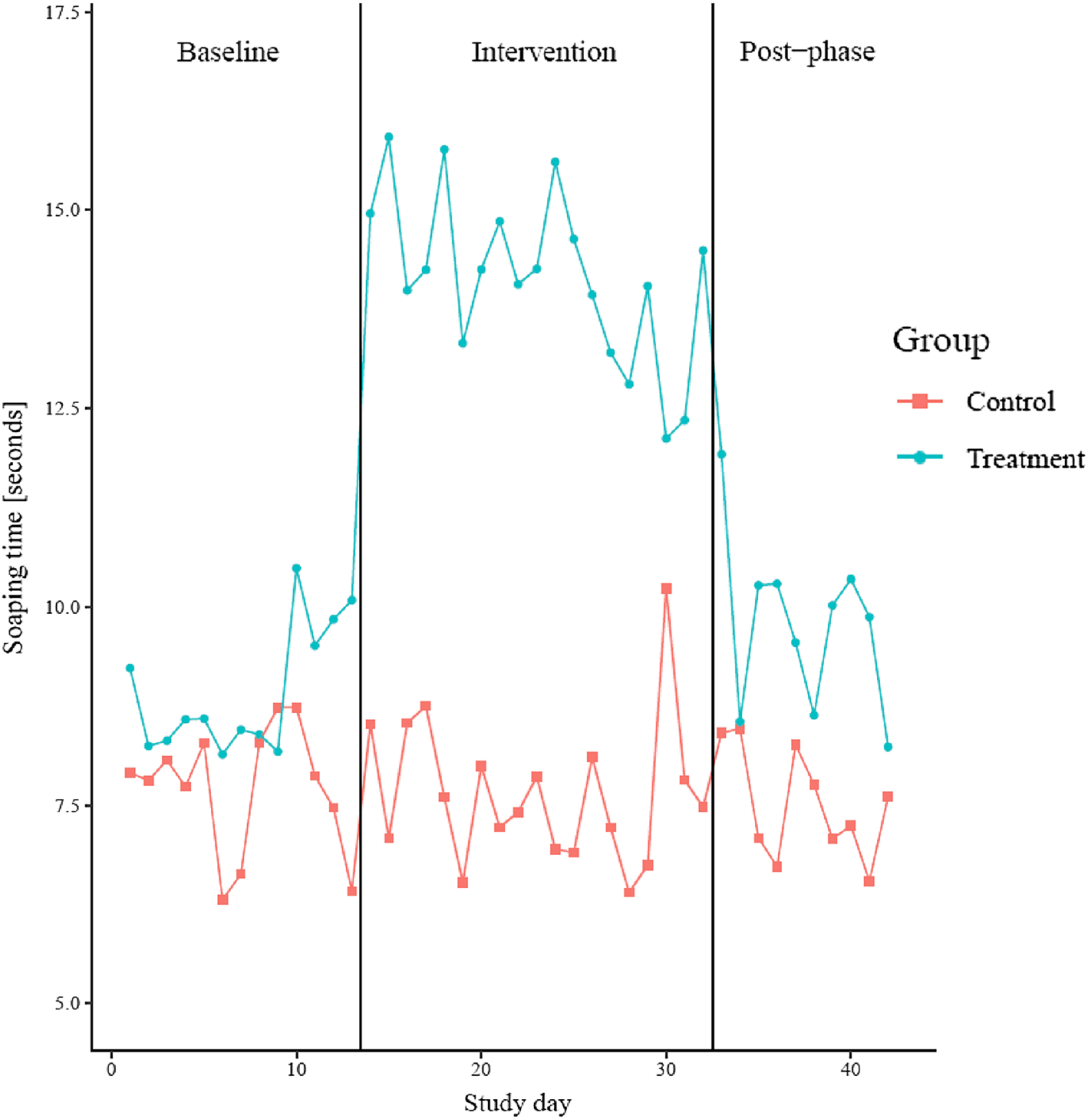
Development of soaping time over time in the treatment and the control group during the baseline, intervention, and post-phase (aggregation of German and Finnish day care centres).

The collected measurement data allows to evaluate the effect of the intervention on handwashing quality and frequency. When the intervention is in place, we observe that children increase the time spent soaping their hands by 62% when the system is in place (p < 0.001). The effect of the intervention on soaping time is shown in Fig. 5. With the activation of the tablets in the treatment group (at the end of the baseline period, two-and-a-half-weeks after the study started), the soaping time increased from an average of 8.45 s across day care centres to 13.75 s in the treatment day care centres. Thus, whereas in the baseline condition not even half of the recommended soaping time was observed (8.45 compared to the recommended 20 s), 69% of the recommended soaping time was observed during the intervention. The effect appeared as soon as the intervention started and remained during the entire intervention phase.

Consolidated results are displayed in the column “Handwashing quality” of Table 2. Investigating results for each day care centre and each stratum separately shows similar results regarding soaping time. While the effect of the experimental phase on the soaping time is not significant, i.e., no change in the control group between the phases, the intervention led to a statistically significant (p < 0.001) increase of 5.30 s (+ 62%) in soaping time. The effect persists throughout the entire intervention period of 4 weeks (see Fig. 5). In the phase without the feedback (post-phase), we observe that soaping time declines, but it is still longer than in the baseline phase (+ 1.22 s compared to the baseline phase, p < 0.001).

**Table 2:** Main experimental outcomes, including the effect of the treatment and the experimental phase on the handwashing quality (measured by soaping time in seconds) and on the handwashing frequency of the children in the day care centre.

	Handwashing quality (soaping time)	Handwashing frequency
*Intervention phase*		
* Treatment effect (*${\beta }_{2}$*)*	+ 5.297*** (0.884)	+ 0.565 (0.900)
* Phase effect (*${\beta }_{1}$*)*	+ 0.063 (0.816)	− 0.878 (0.851)
*Post-phase*		
* Treatment effect (*${\beta }_{4}$*)*	+ 1.221*** (0.349)	− 0.278 (0.694)
* Phase effect (*${\beta }_{3}$*)*	− 0.054 (0.302)	− 0.644 (0.558)
*Overall intercept*	8.447	2.999
*Observations*	13,466	141
*R*^2^	0.060	0.178

The table displays the effects on soaping time (in seconds). Standard errors are reported in parentheses, adjusted for clustering at the day care centre level. *, **, and *** indicate significance at the 5%, 1% and 0.1% level, respectively.

Furthermore, we evaluate the effect of the intervention on the handwashing frequency. The coefficients are presented in the column “Handwashing frequency” of Table 2. We observe a positive trend for the daily handwashing frequency in the baseline phase in both groups and in the intervention phase for the treatment group. However, for both effects, we were unable to demonstrate their statistical significance in our experiment. Thus, children did not substitute higher handwashing quality with lower handwashing quantity during the treatment. Thus, data shows that the intervention considerably increased the quality, but not the frequency of handwashing.

## Discussion

While hand hygiene has been an issue in academia for some time, it has only recently gained new momentum in the public debate due to the pandemic^40^. Despite the new awareness of hand hygiene among the public and the attention given to handwashing in families, the handwashing of children we experienced in the baseline phase of our experiment showed great potential for improvement. The digital intervention introduced in this study increased the soaping time of handwashes performed by children in the treatment day care centre. Notably, the positive effect of our intervention unfolded immediately at the beginning of the intervention. Although the clusters had different baseline characteristics to some extent (e.g., number of children in each day care centre and water extraction per child, see Table 1), our field study proved the effectiveness of the intervention due to the large effect size of 62%. The effect materialises immediately at the onset of the digital intervention (i.e., already at the first exposure to the intervention), which implies that it is effective even in places that are visited only once. Since the post phase was rather short, statements regarding the development of behaviour for the time after the intervention are to be treated with care. The focus of our experiment was on the examination of effects during the intervention and deriving unambiguous and robust results of the treatment effect while the intervention was in place.

The measurement furthermore allows statements about changes regarding handwashing frequency. No statistically significant changes of the handwashing frequency are observable during the experiment. However, we do see a positive trend of the handwashing frequency in the baseline phase and for the intervention (not statistically significant). One plausible argument for the positive point estimate in the baseline phase is that the educational video and the automatic water taps made children and staff more aware of the importance of handwashing in the baseline phase, an effect that wore off over time. The positive point estimate for the intervention can be explained by the reward animation that made handwashing more interesting for children, potentially increasing children’s handwashing frequency.

Our remote system and information systems in general enable the performance of long-term field experiments, which can complement existing lab and observational data^41^. The digital system allows us to directly measure soaping time, a key parameter of handwashing behaviour. The findings have high internal validity, as our system enables nonintrusive, remote measurements that are objective, person-independent, and are not perceived as controlling by the children. Existing studies which investigate the effectiveness of hand hygiene interventions among children do so by looking at absences and infection rates, since those are tracked in educational institutions anyways^42^, however drawing conclusions about handwashing behaviour is equivocal. Studies that directly measure hand hygiene mostly happen in healthcare settings with adults, where hand hygiene compliance audits happen anyhow^42^.

Our experiment confirmed the relevance, scalability, and effectiveness of our digital system in several ways. Five out of the six contacted day care centres were interested to participate in our experiment, despite the usual shortage of personnel and general capacity issues in day care centres. The willingness to take on a side project confirms the interest and the demand of day care centres in hand hygiene and digital technologies. Day care centre management and teachers seemed to highly value support for caretakers regarding hand hygiene.

The experiment confirmed the large impact of our intervention over the course of the 4-week intervention phase. As the effect of the intervention on handwashing was visible immediately after launching it, the intervention is suitable not only for installations in day care centres but also for washbasins, where children come by only once to increase soaping time, spark interest and potentially a conversation with accompanying adults. The impact of the system is high, considering that improved hand hygiene leads to fewer infections^11,12^ which in turn reduces absent days not only of children but also of parents 10.1111/apa.16628﻿ caused by the need to care for their sick child. Importantly, the system does not require any attention or action from the day care centre staff.

While our digital system is promising regarding solving the long-term aim of researchers and practitioners to effectively teach children good hand hygiene, our study has noteworthy limitations. While the population of the study's day care centres is sufficient to achieve the sample size calculated with the power test, the study, with only 4 kindergartens, could be significantly expanded in size. Furthermore, the clusters in the experiment vary in the sense that the number of children in each day care centre differed. The hours that children spend in day care centres per day also differ across clusters, explaining differences in the number of daily water extractions per child.

Future research should investigate whether children's behaviour change persists in the long term and in places where there is no such intervention in place. Although the intervention is beneficial even without habit formation, long lasting behaviour change would greatly increase its value.

We can only speculate which behaviour change mechanisms were key to the success of our intervention. The salience of the display and thus the attention to the activity of handwashing is one possible influencing factor. The fun factor of handwashing, an everyday task that is usually not very exciting might be increased by the animation. To strengthen long-term engagement, the intervention could include regular changes of the reward, which can easily be implemented remotely. Increases in self-efficacy^43^ and intrinsic motivation may also play a role in children’s behaviour change. Future research should investigate mechanisms moderating the behaviour change to ultimately enable to transfer the large impact of our intervention to other applications and target groups. Since the effect of real-time feedback has also proven successful with adults^26^, our digital system could be used as a starting point to develop a system that aims at improving hand hygiene of adults, e.g. in hospitals or nursing homes to protect vulnerable groups for whom infections can be life-threatening.

Overall, our study showed how real-time instructions and symbolic rewards can be implemented in a viable digital solution and how they can guide and support children in successfully performing everyday tasks that are often sources of conflict between caregivers and children. We deliberately focused on handwashing for children because hand hygiene is critical to health, children are especially vulnerable, and sick children greatly impact their parents’ lives. Transferring the principles of our digital intervention to other everyday tasks performed by children, e.g., teeth brushing, opens a powerful way to teach children in the modern era.

## Data Availability

The datasets generated during and analysed during the current study are available from the corresponding author on reasonable request.
